# Refinement of 1p36 Alterations Not Involving *PRDM16* in Myeloid and Lymphoid Malignancies

**DOI:** 10.1371/journal.pone.0026311

**Published:** 2011-10-21

**Authors:** Francois P. Duhoux, Geneviève Ameye, Virginie Lambot, Christian Herens, Frédéric Lambert, Sophie Raynaud, Iwona Wlodarska, Lucienne Michaux, Catherine Roche-Lestienne, Elise Labis, Sylvie Taviaux, Elise Chapiro, Florence Nguyen Khac, Stéphanie Struski, Sophie Dobbelstein, Nicole Dastugue, Eric Lippert, Frank Speleman, Nadine Van Roy, An De Weer, Katrina Rack, Pascaline Talmant, Steven Richebourg, Francine Mugneret, Isabelle Tigaud, Marie-Joëlle Mozziconacci, Sophy Laibe, Nathalie Nadal, Christine Terré, Jeanne-Marie Libouton, Anabelle Decottignies, Miikka Vikkula, Hélène A. Poirel, on behalf of the Groupe Francophone de Cytogénétique Hématologique (GFCH) and of the Belgian Cytogenetic Group for Hematology and Oncology (BCG-HO)

**Affiliations:** 1 Center for Human Genetics, Cliniques Universitaires Saint-Luc, Université Catholique de Louvain, Brussels, Belgium; 2 Centre de Génétique humaine, Centre Hospitalier Universitaire, Liège, Belgium; 3 Centre Hospitalier Universitaire, Nice, France; 4 Center for Human Genetics, Katholieke Universiteit Leuven, Leuven, Belgium; 5 Centre Hospitalier Universitaire, Lille, France; 6 Hôpital Arnaud de Villeneuve, Montpellier, France; 7 Unité de cytogénétique Hématologique, Groupe Hospitalier Pitié-Salpétrière, Paris, France; 8 Hôpital Purpan, Toulouse, France; 9 Centre Hospitalier Universitaire, Bordeaux, France; 10 Centre for Medical Genetics, Ghent University Hospital, Ghent, Belgium; 11 Institut de Pathologie et de Génétique, Gosselies, Belgium; 12 Centre Hospitalier Universitaire, Nantes, France; 13 Centre Hospitalier Universitaire, Dijon, France; 14 Centre Hospitalier Lyon Sud, Lyon, France; 15 Institut Paoli-Calmettes, Marseille, France; 16 Centre Hospitalier Universitaire Hôpital Nord, Saint-Etienne, France; 17 Centre Hospitalier de Versailles, Versailles, France; 18 de Duve Institute, Université Catholique de Louvain, Brussels, Belgium; Brunel University, United Kingdom

## Abstract

Fluorescence *in situ* hybridization was performed to characterize 81 cases of myeloid and lymphoid malignancies with cytogenetic 1p36 alterations not affecting the *PRDM16* locus. In total, three subgroups were identified: balanced translocations (N = 27) and telomeric rearrangements (N = 15), both mainly observed in myeloid disorders; and unbalanced non-telomeric rearrangements (N = 39), mainly observed in lymphoid proliferations and frequently associated with a highly complex karyotype. The 1p36 rearrangement was isolated in 12 cases, mainly myeloid disorders. The breakpoints on 1p36 were more widely distributed than previously reported, but with identifiable rare breakpoint cluster regions, such as the *TP73* locus. We also found novel partner loci on 1p36 for the known multi-partner genes *HMGA2* and *RUNX1*. We precised the common terminal 1p36 deletion, which has been suggested to have an adverse prognosis, in B-cell lymphomas [follicular lymphomas and diffuse large B-cell lymphomas with t(14;18)(q32;q21) as well as follicular lymphomas without t(14;18)]. Intrachromosomal telomeric repetitive sequences were detected in at least half the cases of telomeric rearrangements. It is unclear how the latter rearrangements occurred and whether they represent oncogenic events or result from chromosomal instability during oncogenesis.

## Introduction

Chromosomal band 1p36 is a cytogenetic band of 28 megabases (Mb) located at the telomeric part of the short arm of chromosome 1. This region is primarily known for its constitutional deletions, which lead to polyvisceral and polymorphic defects due to a contiguous gene syndrome [1], [2]. Non-homologous end-joining repair (NHEJ) seems to play a major role in most constitutional 1p36 rearrangements [3]. Acquired 1p36 alterations are frequent in various solid tumors, especially in neuroblastomas in which deletions of 1p36 are commonly encountered and correlated with an unfavorable outcome [4]. Loss of heterozygosity (LOH) of the 1p36 region, found in 20 to 40% of these tumors, suggests the presence of one or more tumor suppressor genes. However, the genes targeted by this prognostically relevant chromosomal aberration remain unknown, raising the question of the possible implication of micro-RNAs (miRNAs) [5], [6].

1p36 alterations are recurrent in hematological malignancies and present in various forms. The most frequent abnormalities in both myeloid and lymphoid neoplasias are unbalanced chromosomal rearrangements, usually described as add(1)(p36) [7], leading to terminal 1p36 deletions. However, other clonal rearrangements have been reported, including reciprocal translocations (35%), unbalanced rearrangements (58%) and deletions (7%) [8]. Cytogenetic 1p36 rearrangements are frequent in non-Hodgkin lymphomas (NHL) (12% of cases) [9], and 1p36 deletions are encountered in roughly 20% of follicular lymphomas (FL) and correlated with poor outcome [10]. There are no data on the frequency of 1p36 rearrangements in myeloid malignances.

As 1p36 alterations are frequent in both solid and hematological tumors, common oncogenic mechanisms may be involved, affecting tumor suppressor genes in the case of deletions, oncogenes in the case of balanced translocations and a combination of both in the case of unbalanced rearrangements. The confirmed or putative tumor suppressor genes mapped to 1p36 [11] include *CDK11A* (*CDC2L2*) and *CDK11B* (*p58* or *CDC2L1*) [12], *TNFRSF1B* (*TNFR2*) [13], *ID3* [14], *NBL1* (*DAN*) [15], *PAX7* [16], *TP73* [17] and *RUNX3* [18]. There are also oncogenes, including *SKI* [19] and *PRDM16* [20]. Other 1p36 genes, like *MDS2*, are involved in chromosomal translocations in hematological malignancies, but their role is still unknown [21].

Following the example of *PRDM16*, which we found to be recurrently rearranged in 31/61 acute myeloid leukemias (AML) and myelodysplastic syndromes (MDS) with cytogenetic 1p36 rearrangements (Duhoux FP *et al*, submitted manuscript), our aim was to identify other recurrent rearrangements on 1p36. While array-Comparative Genomic Hybridization (CGH) data have already been published in certain hematological malignancies [22], they did not focus on 1p36. Moreover, arrays are not the preferred technique for the characterization of balanced and/or telomeric rearrangements. We therefore decided to characterize a series of 81 hematological malignancies using a contig of approximately 120 bacterial artificial chromosomes (BAC) dispersed over 28 Mb [mean resolution of 250 kilobases (kB)] and 20 fosmids to study more precisely specific 1p36 loci.

## Materials and Methods

### Study material

Cytogenetic pellets from 81 patients with hematological malignancies displaying 1p36 alterations not involving *PRDM16* were included in the present study. They were collected from 18 different centers in the frame of a joint study of the Groupe Francophone de Cytogénétique Hématologique (GFCH) and of the Belgian Cytogenetic Group for Hematology and Oncology (BCG-HO).

### Ethics statement

The Ethics Committee of Saint-Luc Hospital approved this study (2007/27-28-29). Such retrospective studies on tissue samples, some of which were stored for many years, do not fall under the scope of the Belgian law (2004) on experimental studies in human beings. Experimentation on residual tissue already stored, as it is the case in this protocol, is allowed if the Ethics Committee gives a positive opinion. In this case, the Ethics Committee waved the need for written patient consent and gave a positive opinion to the investigator.

### Conventional cytogenetic analysis

Karyotyping was performed on bone marrow, peripheral blood or lymph nodes by the referring center following local procedures. All karyotypes were reported according to ISCN 2009 [23] and centrally reviewed twice according to the GFCH and BCG-HO rules.

### Fluorescence in situ hybridization (FISH)

Dual-color FISH was performed centrally (Brussels, Belgium) on fixed nuclei and metaphases. Commercially available probes were obtained from Abbott Molecular (Ottignies/Louvain-la-Neuve, Belgium), Kreatech (Amsterdam, The Netherlands) and Dako (Heverlee, Belgium). Sub-telomeric probe TelVysion 1p (Abbott Molecular, Ottignies/Louvain-la-Neuve, Belgium) and pan-telomeric probe Star*FISH© (Cambio, Cambridge, UK) were used in selected cases. Specific BAC or fosmid probes selected from the Ensembl (www.ensembl.org) or UCSC (genome.ucsc.edu) databases were obtained from the BACPAC Resources Center at the Children's Hospital Oakland Research Institute, Oakland, CA, USA. DNA extractions, labeling and hybridizations were performed as previously reported [24]. All hybridized metaphases were captured on a Zeiss Axioplan 2 microscope (Zeiss, Zaventem, Belgium), and analyzed with the Isis software (Metasystems, Altlussheim, Germany). Metaphase FISH, which was performed in all cases, was complemented by interphase FISH (up to 200 nuclei per sample) in selected cases. In certain cases, the same slides were reused up to 3 times (protocol available on request).

### Cell lines for telomere length quantification

Using cell lines whose telomere length had been previously quantified by Southern Blot-based TRF (Telomere Restriction Fragment) (LB37 +/− 1.5 kb, HeLa +/− 2 kb, SW39 +/− 3.5 kb, LB23 +/− 5 kb, MG63 +/− 7 kb and MZ2 +/− 15 kb) [25], we determined that the minimal telomere length that was detectable with the pan-telomeric Star*FISH probe in our hybridization conditions was of approximately 3.5 kb.

### Single nucleotide polymorphism (SNP) arrays

A DNA sample from patient 142 was analyzed using the GeneChip Human Mapping 250K NspI (Affymetrix Inc., Santa Clara, CA, USA) according to manufacturer's instructions. Data acquisition was performed using the Genotyping Console version 4.1 (Affymetrix).

## Results

Patients' characteristics are described in Table S1. Among the 81 patients, there were 38 myeloid, 42 lymphoid and 1 undifferentiated hematological malignancy. 27 had balanced translocations, 39 unbalanced rearrangements and 15 telomeric rearrangements. The median age of the patients was relatively similar between the 3 subgroups: 62 years in the group of balanced translocations (range 4–81), 56 in the group of unbalanced rearrangements (range 1–87) and 62 in the group of telomeric rearrangements (range 13–77). There were 2 pediatric (<18 years) cases in the balanced, 6 in the unbalanced and 2 in the telomeric subgroups.

### Balanced translocations

**Figure 1:**
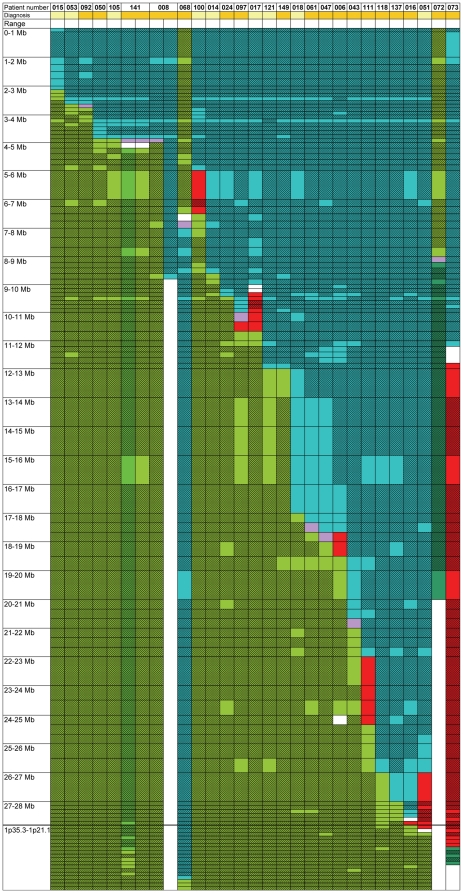
Balanced translocations. Each column represents a case, each line represents a BAC or fosmid probe. 1 Mb intervals on 1p36 are represented at the same size. Diagnoses are highlighted in gold in myeloid cases and in light yellow in lymphoid cases. The results of the used BAC probes are represented in unshadowed boxes, inferences are represented in shadowed boxes. Cells are painted green, if the probe hybridizes to the der(1), blue if the probe hybridizes to another chromosome, purple if the probe is split between the der(1) and another chromosome, and red if the probe is deleted. Additional details are available in Table S2.

Among the 27 patients with balanced translocations, 18 had myeloid malignancies [9 AML, 6 MDS, 1 chronic myelogenous leukemia (CML), 1 polycythemia vera (PV) and 1 myelofibrosis (MF)] and 9 lymphoid malignancies [3 B-cell precursor acute lymphoblastic leukemias (ALL), 3 chronic lymphocytic leukemias (CLL), 1 Burkitt-like lymphoma, 1 marginal zone lymphoma (MZL) and 1 T-cell lymphoma (TCL)]. Results of FISH analysis are depicted in Figure 1 and in Table S2.

In total, the breakpoints on 1p36 were spread over more than 25 Mb. In 8 cases, microdeletions of various sizes were detected near the breakpoints, while in other cases the FISH pattern (split within a BAC) suggested the absence of breakpoint-associated microdeletions at a resolution of approximately 100 kb. Fourteen different partner chromosomes were identified in the 20 cases in which they were identifiable.

**Figure 2:**
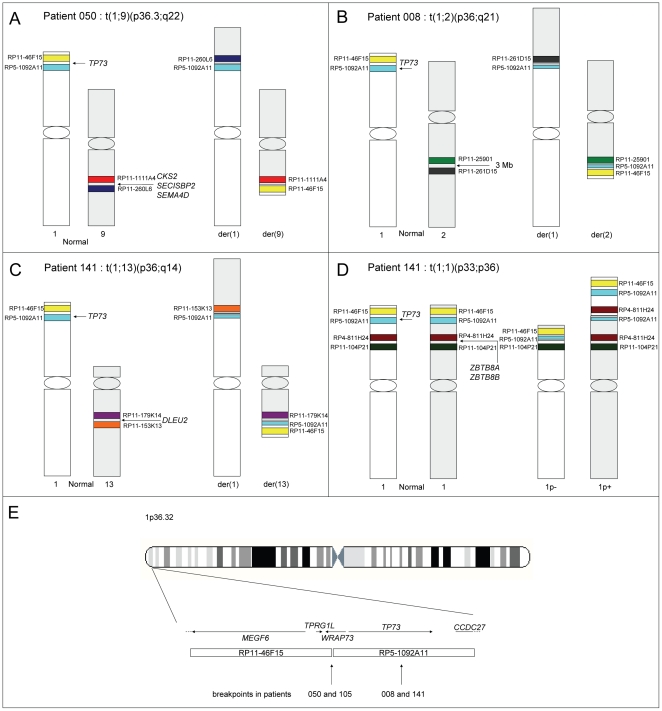
*TP73* rearrangements. Schematic representation of the position of BAC probes and loci of interest in patients 050, 008, 141 and 105 (patients with *TP73* involvement), adapted from www.ensembl.org. Due to lack of material, the partner locus could not be determined in the case of patient 105. Of note, in patients 050 and 105, given the FISH resolution, we could not rule out an involvement of the *WRAP73* locus, which is a member of the WD repeat family, involved in a variety of cellular processes, including cell cycle progression, signal transduction, apoptosis, and gene regulation [53].

No obvious hotspot could be identified except for 2 recurring breakpoints, defined as 2 or more cases harboring the same breakpoint. The first occurred within the *TP73* locus, in which two breakpoint patterns were observed (Figure 2): the breakpoint was either between probes RP11-46F15 and RP5-1092A11 in patients 050 and 105, or within the latter probe in patients 008 and 141. In patient 050, the BAC pattern on 9q22.2 suggested an involvement of the *CKS2* (a cell cycle regulator [26]), the *SECISBP2* or the *SEMA4D* locus. Due to lack of material, the partner locus of patient 105 could not be identified. In patient 008, the partner locus of *TP73* was located in a 3 Mb region on 2q22. Finally, in patient 141, there were 2 balanced translocations, both affecting the *TP73* locus, emphasizing that *TP73* is a recurrent target of 1p36 alterations: in a first subclone, it was rearranged with a region on 13q14.2 comprising the *DLEU2* locus, while in a second subclone, the partner of *TP73* was located on 1p35.1 in a region containing only the *ZBTB8A* and *ZBTB8B* loci.

The second recurrently involved region on 1p36 (patients 121 and 149), was found between probes RP11-56N19 and RP11-929P4, an interval of 722 kb which contains, among others, *TNFRSF8* (a positive regulator of apoptosis) and *TNFRSF1B*. In patient 149 (a MF), we identified the partner locus as *HMGA2* on 12q14.3 using break-apart probes RP11-125M17 and RP11-427K2, suggesting a novel partner locus on 1p36 for this multi-partner gene whose disruption and aberrant expression has been observed in various myeloid malignancies [27].

In addition, we found a novel partner locus on 1p36 for *RUNX1* in a case of MDS (patient 118), as shown with break-apart probes RP11-8P19 and RP11-12N9. In this latter case, the breakpoint on 1p36 was located in a region of 1.2 Mb between probes RP3-465N24 and RP11-492M19. The known *RUNX1* partner loci *PRDM16* [28]and *YTHDF2* [29]on 1p36 and 1p35, respectively, were excluded.

### Unbalanced, non-telomeric rearrangements

**Figure 3:**
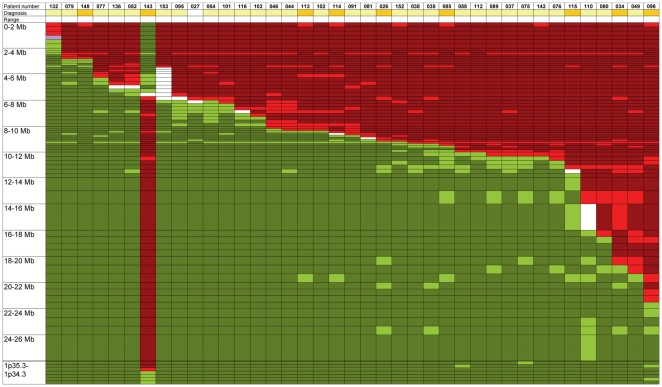
Unbalanced, non-telomeric rearrangements. Each column represents a case, each line represents a BAC or fosmid probe. 2 Mb intervals on 1p36 are represented at the same size. Diagnoses are highlighted in gold in myeloid cases and in light yellow in lymphoid cases. The results of the used BAC probes are represented in unshadowed boxes, inferences are represented in shadowed boxes. Cells are painted green if the probe hybridizes to the der(1), purple if the probe signal is weaker on the der(1) than on the normal chromosome 1, and red if the probe is deleted. There was one case of interstitial deletion (case 143) that was included in this subgroup as it did not belong to any of the other two subgroups; it was ordered according to its breakpoint on 1p36. Additional details are available in Table S3.

The 39 patients with unbalanced rearrangements included 9 myeloid disorders (7 AML, 1 MDS and 1 CML), 29 lymphoid malignancies [2 B-cell precursor ALL, 1 lymphoproliferative syndrome (LPS), 17 FL, 5 diffuse large B-cell lymphomas (DLBCL), 2 MZL, 1 T-cell ALL and 1 peripheral T-cell lymphomas (PTCL)] and 1 acute undifferenciated leukemia (AUL). There were 27 B-cell lymphoproliferations, of which 19 presented with t(14;18)(q32;q21) (16 FL and 3 DLBCL). Results of FISH analysis are depicted in Figure 3 and in Table S3.

The cytogenetic presentation was heterogeneous (Table S1). Besides der(1)t(1;1)(p36;q12–22) (N = 10), the partner chromosome could only be identified in 6 cases exhibiting 6 different chromosomal partner regions. In all but one case, the unbalanced rearrangement led to the deletion of the terminal portion of the 1p36 band and to the addition of supplementary material beyond the breakpoint. The size of the 1p deletion ranged from 1.8 to 21 Mb. Out of the 39 cases, 19 had a breakpoint on 1p36 clustered in a region of 2.4 Mb between RP11-902J21 on 1p36.23 and RP11-420G9 on 1p36.22. Of these 19 above-mentioned cases, 15 were derived from the B-cell lineage and 9 harbored a t(14;18)(q32;q21). FISH in one case of FL transformed into a DLBCL and lacking a t(14;18)(q32;q21) (case 142) showed a der(1)t(1;6)(p36.22;p22.2) that resulted in a deletion of 1p36 telomeric to probe RP11-177N11 on 1p36.22 and a partial 6p trisomy ranging from 6p22.2 to 6pter, as determined by GeneChip Human Mapping 250K Array Set (data not shown).

### Telomeric rearrangements

**Figure 4:**
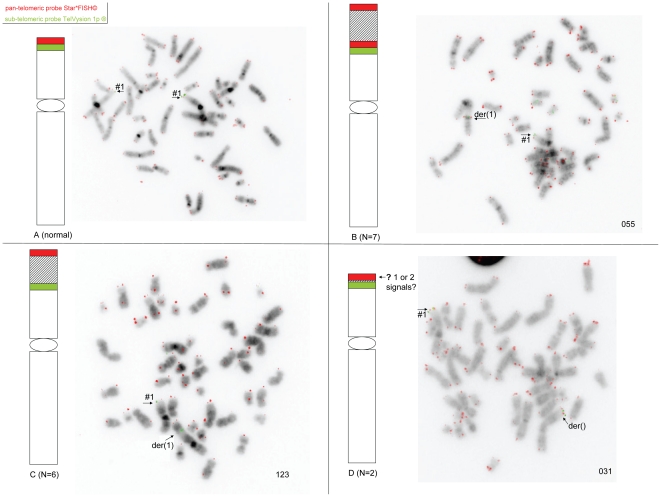
Telomeric rearrangements. Schematic representation of the probes used and of the findings in the cases of telomeric rearrangements. The sub-telomeric probe TelVysion 1p ® is represented in green, the pan-telomeric probe Star*FISH© in red. Material from chromosome 1 is represented in white and the additional material is shaded. A: normal pattern; B: the sub-telomeric and pan-telomeric probes are retained in a centromeric position to the additional material (7 cases); C: only the sub-telomeric probe is retained in a centromeric position to the additional material (6 cases); D: the sub-telomeric probe is present and the presence of the pan-telomeric probe cannot be evaluated due to the resolution of the FISH probes (2 cases): given the shortness of the additional material, it is impossible to assess whether the signal seen with the pan-telomeric probe corresponds to the signal seen in all cases at the telomeric end of the derivative chromosomes, or whether it corresponds to the juxtaposition of this telomeric signal with an intra-chromosomal signal. Additional FISH results are available in Table S4.

There were 15 patients with rearrangements located at the extreme telomeric end of chromosomal band 1p36.33, as identified with the sub-telomeric 1p probe and the pan-telomeric probe. The additional chromosomal material was located (i) telomerically to the canonical repetitive telomeric sequences on the derivative chromosome 1p in at least 7 cases (Figure 4B), (ii) between the sub-telomeric 1p region and the first 3.5 kb of the repetitive telomeric region in 6 cases (Figure 4C), (iii) while in 2 further cases, the possible presence of intrachromosomal telomeric repetitive sequences could not be assessed due to an insufficient length of the additional material (Figure 4D). Eleven out of 15 patients from this subgroup had myeloid [5 AML, 2 MDS, 1 PV, 1 MF, 1 chronic eosinophilic leukemia (CEL) and 1 Fanconi's anemia (FA)] and 4/15 had lymphoid malignancies [1 B-cell ALL, 2 mantle cell lymphomas (MCL) and 1 TCL]. Although the bone marrow was morphologically normal, the case of FA (patient 125) had acquired a subclonal add(1)(p36) in addition to a previously identified gain of the long arm of chromosome 1, suggesting the clonal evolution towards a hematological malignancy. Results of FISH analysis are depicted in Figure 4 and in Table S4.

### Associated chromosomal aberrations

The identity of the partner chromosomes was heterogeneous in the cases in which they could be identified. The rearrangement affecting 1p36 was the only detected rearrangement in 12 cases. They were mainly myeloid malignancies (only 1 CLL). A complex karyotype (≥3 abnormalities) was found in 19/27 (70%) balanced, 29/39 (74%) unbalanced and 8/15 (53%) telomeric rearrangements. Very complex karyotypes (≥5 abnormalities) were observed in only 7/27 (26%) balanced rearrangements, while they were observed in 20/39 (51%) unbalanced and 6/15 (40%) telomeric rearrangements. Of note, among the 16 simple karyotypes in the subgroups of unbalanced and telomeric rearrangements, 5 were der(1)t(1;1)(p36;q12–22) (31%).

## Discussion

Structural rearrangements at 1p36 are relatively frequent in solid tumors [4] and in hematological malignancies [8], especially in NHL [9], [22], [30], [31], [32]. We confirmed the heterogeneity of 1p36 rearrangements. We showed that the breakpoints on 1p36 are more widely distributed than previously reported [8] [33] and identified new loci of interest.

1p36 deletions are observed in myeloid malignancies. In our series, there were AML and MDS in all 3 identified rearrangement subgroups. Prior studies have shown a high proportion of allelic loss on 1p in patients with MDS (13%) [34] and in patients with MDS evolving into AML (30%) [35]. The 1p36 rearrangements in myeloid disorders in our series were more frequently balanced and/or telomeric than in lymphoid disorders, as well as more often isolated, emphasizing the oncogenic relevance of the event on 1p36. On the other hand, the complexity of unbalanced rearrangements in lymphoid disorders may reflect genomic instability.

It is suspected that the loss of tumor suppressor genes on 1p36 contributes to oncogenesis [36]. In our series, *TP73*, one of the potential 1p36 candidate tumor suppressor genes in B-cell lymphomas [37]–[38], was deleted in 35 out of 39 cases with unbalanced non-telomeric rearrangements. The involvement of the *TP73* locus in 4 cases of balanced translocation in our series (3 myeloid and 1 lymphoid cases) might indicate that this gene is targeted by at least some 1p36 rearrangements, as some balanced translocations may lead to inactivation of tumor suppressor genes [39], a mechanism that might have occurred in these cases.

In addition to *TP73* and *PRDM16*, recently shown to be rearranged with various partner genes in myeloid as well as lymphoid malignancies (Duhoux FP *et al*, submitted manuscript), we identified 3 other interesting genes, likely involved in the pathogenesis of hematological malignancies: *PRKCZ*, *RERE* and *AJAP1*. *PRKCZ* and *RERE* were the only genes at the 1p36 breakpoint in 2 CLL patients, respectively in a t(1;4)(p36;q25) (case 015) and in an isolated t(1;13)(p36;q13) (case 014). While balanced translocations in CLL are infrequent, their presence is an adverse prognostic factor [40]. *PRKCZ* is a putative oncogene, as it encodes a member of a family of serine/threonine kinases involved in proliferation, differentiation and secretion [41], while *RERE* may be a a putative tumor suppressor gene because of its involvement in the control of cell survival [42] and because it is the most centromeric locus disrupted in 3 cases of unbalanced rearrangements (cases 044, 113 and 102, i.e. respectively 1 LPS, 1 AML and 1 FL).

*AJAP1*, a putative neuroblastoma tumor suppressor gene [43], was the most centromeric disrupted gene in a case of T-cell ALL (case 136) and in a case of MZL (case 052) with unbalanced rearrangements. All the loci telomeric to *RERE* and *AJAP1* tested in these cases were deleted, raising questions about the role played by these genes.

The common impact of unbalanced rearrangements may be the terminal 1p36 deletion, as there was no obvious recurrent chromosomal partner in these cases. It is therefore logical to look for one or more candidate tumor suppressor gene(s) in the deleted regions.

The minimal deleted region, deleted in all unbalanced cases but one, is the terminal subtelomeric region of 1.8 Mb. Of note, a relevant locus in the centromeric of this region is the purported tumor suppressor gene *CDK11B*, a kinase gene essential for cell cycle control shown to be deleted in NHL [12].

The *TNFRSF14* locus on 1p36.32 (located at 2.4 Mb from the 1p telomere) was deleted in all our 18 cases of FL. This locus was recently shown to be deleted or associated with LOH in 20% of FL, and these alterations were associated with worse prognosis. The residual allele was affected by nonsynonymous mutations in 3 out of 5 cases of FL with 1p36 deletions [44].

It is noteworthy that in 9 cases of NHL with t(14;18)(q32;q21), the breakpoints were clustered in a region of 2.4 Mb. The most distal commonly deleted gene in this cluster was *TNFRSF9* (located at 8.0 Mb from the 1p telomere), a gene whose expression in the microenvironment of DLBCL was recently shown to predict overall survival (OS) [45]. Furthermore, FISH alone (case 058) or combined with SNP-arrays (case 142) allowed us to locate the 1p36 deletion in the same 2.4 Mb cluster in 2 cases exhibiting a new subtype of FL [46], characterized by a predominantly diffuse growth pattern, a frequent inguinal location, and the formation of large, localized tumors. These tumors lack the t(14;18)(q32;q21) and display a del(1p36) [47]. As 1p36 deletions are similar in FL with and without typical 18q21/*BCL2* translocations, it raises questions about a common initial oncogenic event in both subtypes of FL.

Terminal 1p36 deletions are frequent and can be underestimated by conventional cytogenetics [22]. The identification of these deletions is clinically important in FL with and without (14;18)(q32;q21), as they have been shown to be correlated not only with shorter OS [48] but also with a higher risk of transformation, independently of International Prognostic Index (IPI) [33]. Furthermore, in our series, there were 1p36 deletions in DLBCL with and without (14;18)(q32;q21), a translocation present in up to 20% of DLBCL [49]. It would therefore be interesting to evaluate the prognostic impact of 1p36 deletions not only in FL, but also in DLBCL. Given the heterogeneity of breakpoints on 1p36, we suggest to use very distal BAC probes, in or near the *TNFRSF14* locus, in order to detect 1p36 deletions in FL.

In our series of cases with 1p36 aberrations, add(1)(p36) was the most frequent abnormality. As the nature of the additional chromosomal material was non recurrent when identifiable, with the exception of the t(1;1)(p36;q12–23), we chose to focus on the breakpoints on 1p36 in these cases and not to characterize the partner loci. Among cytogenetically identified aberrations, the most common was thus the t(1;1)(p36;q12–23) (12 cases). This aberration was molecularly heterogeneous and breakpoints on 1p were distributed over 19 Mb, displaying a larger dispersion than previously described [50] (Figure 3 and Table S3). This unbalanced rearrangement, leading to 1p36 deletion and 1q duplication, is recurrent in NHL, with an incidence of 3–4% in the t(14;18)(q32;q21) positive FL and DLBCL [50]. A mechanism of homologous recombination is suspected to play a role in this rearrangement. The t(1;1)(p36;q12–23) cases in our series were not restricted to lymphoid malignancies as usually reported but also seen in myeloid malignancies (50% each), raising the question of a common oncogenic mechanism.

1p36 deletions are frequent events in hematological and non-hematological malignancies. At the moment, no consensus tumor suppressor gene has been identified in this region. Whether several tumor suppressor genes are involved, or del(1)(p36) affects small non-coding RNAs, remains elusive. Of note, miRNA *hsa-mir-34a* was included in the deleted region of 18 cases with unbalanced rearrangements, mainly B-cell lymphomas (12/18). *Hsa-mir-34a* is a target of p53 involved in cellular proliferation and apoptosis [6]. Its deletion has already been suggested to play a role in other B-cell lymphoproloferations as well as in neuroblastoma [6], [51].

The 15 very telomeric rearrangements identified by FISH in our study would not have been detected by array-CGH or SNP analysis. There were at least 7 cases with intrachromosomal telomeric sequences. Whether these resulted from the insertion of DNA within the very distal portion of 1p or from chromosome fusion events between 1p and another chromosome end followed by breakage at anaphase (breakage-fusion-bridge cycle) is unknown. If the latter assumption were true, this would reflect chromosomal instability, but why would chromosome fusion events specifically happen at 1p end and not at other chromosome ends? Although we did not detect them in our experimental conditions, we cannot rule out the possibility that intrachromosomal telomeric sequences are also present on other chromosomes. On the other hand, the hypothesis that DNA fragments would be inserted within telomeric sequences of 1p end is in contradiction with the well-established fact that NHEJ is normally prevented at functional telomeres [52]. Hence, it is unclear how these rearrangements at 1p ends occurred and whether they represent oncogenic events or result from chromosomal instability during oncogenesis.

In conclusion, using FISH, we characterized the breakpoints on 1p36 in 81 cases of hematological malignancies with cytogenetic 1p36 alterations not affecting the *PRDM16* locus and showed that the breakpoints were more widely distributed than previously reported. We defined breakpoint cluster regions, especially within or near the *TP73* locus. We refined the terminal 1p36 deletions in FL with and without t(14;18)(q32;q21). Given that 1p36 losses are known to have a prognostic impact in FL, are cryptic in a proportion of cases and are also detected in DLBCL, we suggest to evaluate the prognostic impact of 1p36 deletions in B-cell lymphomas with distal 1p36 BAC probes, independently of the presence of a cytogenetic 1p36 rearrangement. We also found novel partner regions on 1p36 for the known multi-partner genes *HMGA2* and *RUNX1*. The functional impact of the presence of intra-chromosomal telomeric repetitive sequences is unclear. Whether the 1p36 rearrangements are causal or associated with disease progression remains to be studied. Further molecular and functional studies are required to identify the targeted tumor suppressor gene(s), especially in B-cell lymphomas.

## Supporting Information

Table S1 **Patients' characteristics.**
(DOC)

Table S2 **Balanced translocations.**
(XLS)

Table S3 **Unbalanced, non-telomeric rearrangements.**
(XLS)

Table S4 **Telomeric rearrangements.**
(XLS)
