# Correction: Refinement of 1p36 Alterations Not Involving *PRDM16* in Myeloid and Lymphoid Malignancies

**DOI:** 10.1371/annotation/3b5aaa87-72b6-49d3-9ec9-f4a656e12e3a

**Published:** 2011-12-13

**Authors:** Francois P. Duhoux, Geneviève Ameye, Virginie Lambot, Christian Herens, Frédéric Lambert, Sophie Raynaud, Iwona Wlodarska, Lucienne Michaux, Catherine Roche-Lestienne, Elise Labis, Sylvie Taviaux, Elise Chapiro, Florence Nguyen-Khac, Stéphanie Struski, Sophie Dobbelstein, Nicole Dastugue, Eric Lippert, Frank Speleman, Nadine Van Roy, An De Weer, Katrina Rack, Pascaline Talmant, Steven Richebourg, Francine Mugneret, Isabelle Tigaud, Marie-Joëlle Mozziconacci, Sophy Laibe, Nathalie Nadal, Christine Terré, Jeanne-Marie Libouton, Anabelle Decottignies, Miikka Vikkula, Hélène A. Poirel, on behalf of the Groupe Francophone de Cytogénétique Hématologique (GFCH) and of the Belgian Cytogenetic Group for Hematology and Oncology (BCG-HO)

There was an error in the name of the 13th author. The correct name is Florence Nguyen-Khac.

