# Supplementary material for: Refinement of 1p36 Alterations Not Involving PRDM16 in Myeloid and Lymphoid Malignancies
Source: PLoS One. 2011 Oct 21;6(10):e26311. doi: 10.1371/journal.pone.0026311 (PMC3198844; doi:10.1371/journal.pone.0026311)
Supplement: Table S1 — Patients' characteristics. (DOC) [file pone.0026311.s001.doc]

**Table S**1. Patients' characteristics

| **Pt ID** | **Age (yrs)** | **Sex** | **Diagnosis** | **Previous conditions** | **Follow-up from diagnosis (months)** | **Outcome at latest follow-up** | **Karyotype** | **Breakpoint on 1p36 determined by FISH** | **Consequence** | **Other results** |
| --- | --- | --- | --- | --- | --- | --- | --- | --- | --- | --- |
| **Balanced translocations** | | | | | | | | | | |
| 015 | 79 | F | CLL | None | NA | NA | 47,XX,t(1;4)(p36;q25),del(6)(q14-16q22 or q24),+12,add(22)(q13)[7]/46,XX[3] | ↔ G248P84280G9 and G248P85743D3 | Telomeric 1p36 probes on der(4) | RT-PCR: *IGH* rearrangement; FISH: breakpoint on 4q24 |
| 053 | 53 | M | Burkitt-like lymphoma | None | 6 | Deceased | 46,XY,+X,add(1)(p36),add(2)(q12-14),der(3)add(3)(p21)t(3;14)(q27;q12-21),del(7)(q22),del(9)(p13),der(9)t(9;14)(p13;q32),der(14)t(?9;14)(p13;q32),+mar1,+mar2[cp13]/46,XY[3] | ↔ RP11-799N13 and  RP3-395M20 | Telomeric 1p36 probes on der(14) | RT-PCR: *IGH* rearrangement; FISH: *IGH* and *BCL6* rearranged, *MYC* not rearranged |
| 092 | 75 | F | AML (secondary?) | M+ endometrial cancer | NA | NA | 42~46,XX,add(1)(p36),del(3)(p21),add(4)(q21),del(5)(q23q34),del(6)(p21),-7,-8,der(17)t(17;18)(p11.2;q11.2),-18,?hsr(19)(q13.4),-21,-22,+1~5mar[cp3]/42~46,idem,der(2)t(2;?3)(p25;q?21),-del(3)(p21),-add(4)(q21),der(11)t(11;13)(p11.2;q12),-13,-?hsr(19)(q13.4),inc[cp3]/46,XX[3] | ↓ RP3-395M20 | Telomeric 1p36 probes on unidentified chromosome | None |
| 050 | 78 | F | PV | NA | NA | NA | 47,XX,t(1;9)(p36.3;q22),+9[10]/47,XX,t(1;9)(p36;q22),+der(9)t(1;9)[14] | ↔ RP11-46F15 and RP5-1092A11 | Telomeric 1p36 probes on der(9) | FISH: breakpoint on 9q22.2 |
| 105 | 4 | M | B- ALL | None | 97 | Alive | 46,XY,der(1)t(1;1)(?q;p36),del(5)(q13),t(6;12)(?q14;q24)[11]/46,XY[4] | ↔ RP11-46F15 and RP5-1092A11 | Telomeric 1p36 probes on unidentified chromosome | FISH: *ETV6*-*RUNX1* fusion associated with *ETV6* deletion |
| 141 | 72 | F | Relapsed MDS (RAEB-2) - 13.5% blasts | None | 31 after initial diagnosis;  24 after relapse | Alive | 46,XX,del(5)(q13q31~34)[2]/46,sl,t(1;13)(p36;q14)[10]/46,sl,t(1;1)(p33;p36)[3]/46,XX[5] | ↓ RP5-1092A11 | Telomeric 1p36 probes on der(1) or on der(13) | No *FLT3* ITD, no *FLT3* D835 mutation, no *NPM* mutation; FISH: breakpoint on 13q14.2 (*DLEU1* probably rearranged) |
| 008 | 59 | M | AML M4 post MDS | MDS | 24 | Alive | 46,XY,?t(6;16)(p22;p13.?3)[3]/46,XY,t(1;6)(p36;p25)[2]/46,XY,t(1;2)(p36;q21)[2]/46,XY(13] | t(1;2): ↓ RP5-1092A11 for  t(1;6): centromeric to RP4-633I8 | t(1;2): Telomeric 1p36 probes on der(2)  t(1;6): Telomeric 1p36 probes on der(6) | RT-PCR: no *MLL* rearrangement, FISH: no *MLL* nor *CBFB* rearrangement |
| 068 | 62 | M | MDS | CTH + RTH for Hodgkin lymphoma | NA | Deceased | 46,XY,inv(1)(p21p36)[14] | ↓ RP11-780G9 | Telomeric 1p36 probes on telomeric part of der(1), centromeric 1p36 probes on inverted segment of der(1) | None |
| 100 | 4 | F | B-ALL | None | 6 | Alive | 59~62<3n>,XXX,add(1)(p36),-1,-2,-3,der(7)?ins(7;?)(p12;?),-9,-9,-10,-11,-13,+14,-15,-16,-17,-19,-20,+21,-22,+4~6mar[cp20]/46,XX[8] | ↔ RP5-1096P7 and RP11-49J3; ↔ RP1-202O8 and RP11-58A11 | Telomeric 1p36 probes on other chromosome, 1p36 probes between the 2 breakpoints are deleted | RT-PCR: no *MBCR*/*ABL1*, *mBCR*/*ABL*, *ETV6/RUNX1*, *HRX*/*AF4* and *TCF3*/*PBX1* rearrangement |
| 014 | 78 | F | CLL | None | 2 | Alive | 46,XX,t(1;13)(p36;q13)[2]/46,XX[23] | ↔ RP5-1115A15 and RP4-633I8 | Telomeric 1p36 probes on der(13) | None |
| 024 | 65 | F | MDS - 1% blasts | None | NA | NA | 46,XX,der(1)(1qter→q24::1p36→q24::5q1?3→qter),der(5)t(1;5)(p36;q1?3),del(20)(q12)[5]/46,XX[5] | ↔ RP11-315O13 and RP3-510D11 | Telomeric 1p36 probes on der(5) | None |
| 097 | 57 | F | MDS (RARS) - no excess blasts | NA | 55 | Alive | 46,XX,t(1;7)(p3?6;q2?2)[18]/46,XX[2] | ↓ RP11-177N11 | Telomeric 1p36 probes on der(7); 1p36 probe RP11-420G9 deleted; all other centromeric 1p36 probes on der(1) | None |
| 017 | 52 | F | CLL | NA | NA | NA | 47,XX,+12[1]/47,idem,t(1;17)(p36;q22)[6] | ↔ RP3-477M7 and RP11-315O13; ↔ RP11-420G9 and RP11-108G8 | Telomeric 1p36 probes on der(17), 1p36 probes between the 2 breakpoints are deleted | Catovsky index 5/5, *ZAP70*(+), CD38(+); FISH: breakpoint on 17q21.31-2 (*WNT3* probably rearranged) |
| 121 | 50 | F | T-lymphoma* | None | 3 | Deceased | 46,XX,t(1;11)(p3?6;q24),t(3;8)(q27;q23)[10]/46,XX[10] | ↔ RP11-56N19 and RP11-929P4 | Telomeric 1p36 probes on der(11) | RT-PCR: *TCRγ* rearrangement; FISH: *BCL6* rearranged, *MLL* and *MYC* not rearranged |
| 149 | 73 | M | Myelofibrosis | PV | NA | NA | 46,XY,t(1;12)(p36.1;q14)[23] | ↔ RP11-56N19 and RP11-929P4 | Telomeric 1p36 probes on der(12) | FISH: *HMGA2* rearranged |
| 018 | 20 | M | B-ALL | NA | 10 | Alive | 47,XY,+X,t(1;7)(p36;q22),?t(3;9)(q24;p12)[3]/46,XY[7] | ↔ RP11-5P18 and RP11-91D22 | Telomeric 1p36 probes on der(7) | None |
| 061 | 66 | F | AML | NA | NA | NA | 47,XX,t(1;9;22)(p36;q34;q11),+8,i(19)(q?)[15] | ↓ RP11-99C7 | Telomeric 1p36 probes on der(9) | FISH: *MLL*(-), inv(16)(-), *RUNX1*/*RUNX1T1*(-), *BCR*/*ABL1* rearranged |
| 047 | 65 | M | AML | None | 10 | Deseased | 45,XY,der(1)t(1;1)(p36;q25),-5,-5,-9,add(17)(p13),der(21)t(5;21)(q11;q22),+2mar[11]/46,XY[4] | ↓ RP11-261J13 | Telomeric 1p36 probes on other unidentified chromosome | FISH: *RUNX1* not rearranged |
| 006 | 39 | M | Relapsed AML M6 | None | 6 between initial diagnosis and relapse, 4 after relapse | Deceased | 44,XY,add(1)(p36),-2,-3,add(5)(q13),-7,del(12)(p13),del(13)(q11q14),-14,?der(17),+mar1,+mar2[3];94~96,idemx2,+2,+3,+4,-5,+6,-9,add(9)(q22),+10,+10,-del(12)(p13),+13,-der(17),+19,-21,+22,+mar3,+mar3[cp18] | ↔ RP11-99C7 and RP3-394P21 | 1p36 probes telomeric to RP11-99C7 on mar3, 1p36 probes RP11-261J13 and RP11-79D15 deleted, 1p36 probes centromeric to RP3-394P21 on der(1) | None |
| 043 | 81 | F | AML M2 secondary to MDS | MDS | 1 | Deceased | 46,XX,t(1;8;6)(p36;q22;p23),add(17)(p12)[5]/46,idem,del(7)(q22q32)[2]/46,XX[13] | ↓ RP11-367G13 | Telomeric 1p36 probes on der(8) | FISH: *MLL*(-) |
| 111 | 70 | M | AML | NA | NA | NA | 48,XY,+Y,t(1;17)(p36;q11),+der(1)t(1;17)[10] | ↔ RP11-487E1 and RP11-10N16 | 1p36 probes telomeric to RP11-487E1 on der(17), 1p36 probes RP11-132G19, RP1-150O5, RP11-223J15 and RP11-89K16 deleted, 1p36 probes centromeric to RP11-10N16 on der(1) | None |
| 118 | 49 | M | MDS (RTC)  Blast count unknow | NA | NA | NA | 46,XY,t(1;21)(p36;q22)[17]/46,XY[3] | ↔ RP3-465N24 and RP11-492M19 | Telomeric 1p36 probes on der(21) | FISH: *RUNX1* rearranged |
| 137 | 51 | M | AML M0 | None | 3 | Deceased | 41~43,XY,add(1)(p36),-3,+add(4)(p22),t(6;10)(p24;q22),add(7)(q11) or del(7)(q11q33),add(11)(q23),add(12)(p13),-14,-16,add(17)(p11),-18,-20[cp20] | ↔ RP11-492M19 and RP11-696E2 | Telomeric 1p36 probes on other unidentified chromosome | FISH: *MLL* amplification, |
| 016 | 36 | F | CML | NA | NA | NA | 46,XX,t(1;7)(p36;q22),t(9;22)(q34;q11.2)[10] | ↔ RP11-4K3 and RP11-288L9 | 1p36 probes telomeric to RP11-4K3 on der(7), 1p36 probe RP1-159A19 deleted, 1p probes centromeric to RP11-288L9 on der(1) | RT-PCR: *BCR*/*ABL1* type b2a2; FISH: breakpoint on 7q22.1 |
| 051 | 74 | M | MZL | None | 1 | Deceased | 46,XY,del(7)(q22q32)[5]/46,idem,t(1;13)(p36;q14)[2]/46,XY,del(6)(q21)[2]/46,XY[18] | ↔ RP3-465N24 and RP11-442N24 | 1p36 probes telomeric to RP3-465N24 on der(13), 1p probes between RP11-285H13 and RP11-288L9 deleted, 1p probes centromeric to RP11-442N24 on der(1) | IgH CDR III monoclonal rearrangement |
| 072 | 63 | F | Relapsed AML M3 | RTH for BC | 38 after initial diagnosis; NA after relapse | Alive | 46,XX,dup(1)(p22p36),t(4;10)(p13;p14),t(15;17)(q22;q21)[19] | ↓ RP11-902J21 | Telomeric 1p36 probes on telomeric der(1); centromeric 1p36 probes on telomeric and on centromeric der(1) | None |
| 073 | 52 | F | MDS post PV – 1% blasts | PV for 18 years | NA | NA | 44,XX,?dic(1;17)(p36;p11),del(5)(q21q33),dic(10;?)(q24;?),-14[15]/44,idem,-15,+mar[1]/44,idem,-5,+mar[2] | ↔ RP11-874A11 and RP4-811H24 | 1p36 probes telomeric to RP11-874A11 on other unidentified chromosome, 1p probes between RP11-56N19 and RP11-266K22 deleted, 1p probes centromeric to RP4-811H24 twice on 1p (centromeric and telomeric) | *JAK2* V617F mutation; FISH: 17q telomeric probe plays role of der(1) telomere |
| **Unbalanced rearrangements** | | | | | | | | | | |
| 132 | 3 | M | B-ALL | None | NA | NA | 55,XY,der(1)t(1;1)(p36;q12),add(2)(p?),+4,+6,+10,+14,+16,+18,+21x2,+mar[cp16]/63-64,idem,+X,+5,+9,+11,+12,+15,+17,+19,+22[cp2]/46,XX[10] | ↓ G248P8962B4 | Telomeric 1p36 probes deleted | FISH: no *BCR*-*ABL*, *MLL*, nor *ETV6*-*RUNX1* rearrangement |
| 079 | 69 | M | Foll NHL | NA | 15 | Deceased | 47,XY,add(1)(p36)del(1)(q42),-4,?add(5)(p14),-5,-6,-8,?+9,t(14;18)(q32;q21),-16,+6mar[15]/46,XY[3] | ↔ RP3-395M20 and RP11-740P5 | Telomeric 1p36 probes deleted | None |
| 148 | 1 | F | AML | NA | NA | NA | 46,XX,der(1)t(1;1)(p36;q21)[12]/46,XX[14] | ↔ RP3-395M20 and RP11-740P5 | Telomeric 1p36 probes deleted | FISH: no *MLL* rearrangement |
| 077 | 79 | M | Foll NHL | Lymphoplasmocytic lymphoma 3 years before | 4 | Alive | 47,X,-Y,+X,add(1)(p36),del(6)q14-16q25-27),+12,t(14;18)(q32;q21)[8] | ↔ RP11-718D19 and RP11-111O5 | Telomeric 1p36 probes deleted | None |
| 136 | 3 | F | T-ALL* | None | 3 | Deceased | 46,XX,add(1)(p36)[5]/46,XX,add(16)(q24)[2]/46,XX[23] | ↔ RP1-37J18 and RP11-58I24 | Telomeric 1p36 probes deleted | RT-PCR: *BCR*-*ABL1*(-) |
| 052 | 51 | F | MZL | NA | 36 | Alive | 50,XX,add(1)(p36),+3,+3,add(12)(q24),add(15)(q15~22),+18,+mar[19]/46,XX[3] | ↔ RP1-37J18 and RP11-58I24 | Telomeric 1p36 probes deleted | None |
| 143 | 87 | M | MDS (RAEB) | NA | NA | NA | 47,XY,del(1)(p34~36),+8[19]/46,XY[1] | ↔ RP11-58I24 and RP11-49J3 / ↔RP3-437I16 and RP3-357I16 | 1p36 probes telomeric to RP11-58I24 are on der(1), 1p probes between RP11-49J3 and RP3-437I16 are deleted, 1p probes centromeric to RP3-357I16 are on der(1) | None |
| 153 | 49 | F | PTCL* | None | 62 | Alive | 48,XX,add(1)(p36),add(2)(q11),-4,-5,del(6)(q?1),+8,del(9)(q21q34),del(10)(q25) or del(10)(q22q24),inv(11)(p13q21~22),del(10)(q22q24),inv(11)(p13q21~22),add(12)(q24),add(17)(p13),del(20)(q12~13),add(22)(q13),+mar1,+mar2,+mar3[5]/46,XX[17] | ↔ RP11-22L13 and RP11-372C15 | 1p36 probes telomeric to RP11-22L13 are deleted, 1p36 probes centromeric to RP11-372C15 are on der(1) | None |
| 095 | 67 | M | Foll NHL | NA | NA | NA | 46,XY,der(1)t(1;2)(p36;q11),add(1)(q?31),-2,add(3)(q27),t(14;18)(q32;q21),-18,+2mar[6]/46,idem,add(12)(q24)[12]/46,XY,der(1)t(1;2)(p36;q11),add(1)(q?31),-2,add(3)(q27),add(12)(q24),del(13)(q11),t(14;18)(q32;q21)[2]/46,XY[1] | ↔ RP5-1096P7 and RP11-372C15 | Telomeric 1p36 probes deleted | RT-PCR: *IGH*-*BCL2* mcr fusion (+) |
| 027 | 17 | M | AUL | None | 24 | Deceased | 46,XY,add(1)(p36),t(10;18;11)(p12;q22;q21)[3]/46,XY[7] | ↔ RP11-49J3 and RP11-71M19 | Telomeric 1p36 probes deleted | RT-PCR: *MLL*-*AF10*(+) |
| 054 | 60 | M | DLBCL | None | 18 | Alive | 50,X,-Y,+X,idic(1;9)(p36;p12),+i(5)(q10),+7,-8,-9,del(9)(p21),+12,t(14;18)(q32;q21),der(15)t(2;15)(q13;p11),+mar1,+mar2[cp5] | ↔ RP11-49J3 and RP11-372C15 | Telomeric 1p36 probes deleted | RT-PCR: *IGH*-*BCL2* mbr fusion (+) |
| 101 | 83 | M | Foll NHL | NA | NA | NA | 47,XY,+X,add(1)(p36),-5,add(5)(q3?2),t(14;18)(q32;q21),add(19)(q13),del(22)(q12),+mar1[5]/47,idem,-4,+r[9] | ↔ RP11-372C15 and RP11-71M19 | Telomeric 1p36 probes deleted | None |
| 116 | 74 | F | Foll NHL | NA | NA | NA | 49,XX,add(1)(p36),+2,+7+,12,t(14;18)(q31;q21)[5]/50,idem,+16[13]/46,XX[2] | ↔ RP1-202O8 and RP11-780G9 | Telomeric 1p36 probes deleted | None |
| 103 | 68 | F | Foll NHL | NA | NA | NA | 89<4n>,XX,del(X)(q22)x2,add(1)(p36)x3,-4,-4,-5,-5,add(5)(q3?1),del(6)(q1?q2?),+7,-8,-8,del(10)(q24),add(12)(q23),-13,t(14;18)(q32;q21)x2,-15,-15,-19,-19,-22,+5~9mar,inc[14]/46,XX[7] | ↔ RP11-780G9 and RP11-845I16 | Telomeric 1p36 probes deleted | None |
| 046 | 71 | F | MZL | NA | NA | NA | 46,XX,der(1)t(1;1)(p36;q21)[6]/46,sl1,t(3;15)(q25;q15)[5]/46,sl2,add(14)(?q32)[2]/46,XX[13] | ↔ RP11-902J21 and RP11-748N23 | Telomeric 1p36 probes deleted | None |
| 044 | 13 | M | LPS | NA | NA | NA | 47,XY,+X,der(1)t(1;1)(p36;q21)[10]/46,XY[10] | ↔ RP11-748N23 and RP5-1115A15 | Telomeric 1p36 probes deleted | None |
| 113 | 19 | F | AML M1 | NA | NA | NA | 47,XX,add(1)(p36),+8,t(11;19)(q23;p13.3)[19]/46,XX[1] | ↔ RP11-748N23 and RP5-1115A15 | Telomeric 1p36 probes deleted | FISH: *MLL* rearranged |
| 102 | 59 | M | Foll NHL | NA | NA | NA | 49,XY,+X,der(1)t?(1;1)(p36;q21),+3,t(14;18)(q32;q21),+der(18)t(14;18)[5]/49,idem,del(6)(q15q22)[5]/51,XY,+X,der(1),+3,+11,+12,t(14;18),+der(18)t(14;18)[2] | ↔ RP11-748N23 and RP5-1115A15 | Telomeric 1p36 probes deleted | RT-PCR: *IGH*-*BCL2* mbr fusion (+) |
| 114 | 1 | M | AML | +21 | NA | NA | 47,XY,der(1)(1q44→1q31::1p36→1q44),+21c[20] | ↔ RP5-1115A15 and RP3-477M7 | Telomeric 1p36 probes deleted | None |
| 091 | 65 | M | DLBCL | Foll NHL for 8 years | 54 | Alive | 52~53,XY,+X,der(1)add(1)(p36)dup(1)(q3?1q4?2),+7,t(14;18)(q32;q21),+der(18)t(14;18),+21,+mar1,+mar,inc[cp3]/53-54,idem,+2[11]/46,XY[6] | ↔ RP4-633I8 and RP3-477M7 | Telomeric 1p36 probes deleted | RT-PCR: *IGH*-*BCL2* mbr fusion (+), FISH: *IGH*-*BCL2* fusion (+) |
| 081 | 41 | F | Foll NHL | NA | NA | NA | 46-47,XX,der(1)t(1;1)(p36;q21),del(4)(q12q21),+del(6)(q13),del(10)(q22q24),trp(12)(q13q21),t(14;18)(q32;q21)[5] | ↔ RP3-477M7 and RP11-315O13 | Telomeric 1p36 probes deleted | None |
| 026 | 33 | M | AML | None | 5 | Deceased | 43-46,XY,add(1)(p36),ins(3;?)(p23;?),der(5)?t(3;5)(p14;q13),-10,+ins(12;?)(p11;?),-13,+14,-17,-21,-22,+2-3mar[cp4]/46,XY[1] | ↔ RP11-315O13 and RP3-510D11 | Telomeric 1p36 probes deleted | RT-PCR: no AML nor *MLL* fusion transcripts |
| 152 | 56 | M | DLBCL | None | 7 | Alive | 46,XY,add(1)(p36),t(3;10)(q28~29;q22),del(12)(p12p13)[14]/46,XY[6] | ↔ RP11-315O13 and RP3-510D11 | Telomeric 1p36 probes deleted | None |
| 038 | 54 | F | Foll NHL | None | 44 | Alive | 46,XX,add(1)(p36),t(14;18)(q32;q21)[11]/46,XX[3] | ↔ RP3-510D11 and RP13-392I16 | Telomeric 1p36 probes deleted | RT-PCR: *IGH*-*BCL2* fusion (+) |
| 035 | 58 | F | B-cell ALL | CT for BC 1 year before | 31 | Alive | 47,XX,add(1)(p36),t(4;11)(q21;q23),+mar[6]/46,XX[6] | ↔ RP3-510D11 and RP13-392I16 | Telomeric 1p36 probes deleted | RT-PCR: *MLL*-*AF4* (+) |
| 085 | 65 | F | Relapsed AML M5 | MDS | 84 between initial diagnosis and relapse,27 after relapse | Deceased | 46,XX,der(1)t(1;1)(p36;q22)[9]/46,XX[11] | ↔ RP13-392I16 and RP11-558F24 | Telomeric 1p36 probes deleted | None |
| 058 | 46 | M | Foll NHL | Hodgkin lymphoma | NA | NA | 46,XY,der(1)t(1;12)(p36;p12),der(12)(?::12p11.2->12q12::?::12q12->12q24.3)[15]/46,XY[5] | ↔ RP11-892D18 and RP11-177N11 | Telomeric 1p36 probes deleted | FISH: *ETV6* on normal 12, der(1) and der(12); breakpoint on 12p13.1 (*ATF7IP* probably rearranged) |
| 112 | 52 | M | DLBCL | Foll NHL 21 months before | 25 | Alive | ?49,XY,add(1)(p36.3),+5,+i(6)(p10),t(14;18)(q32;q21),inc[1] | ↔ RP11-892D18 and RP11-177N11 | Telomeric 1p36 probes deleted | RT-PCR: *IGH*-*BCL2* fusion (+), FISH: *BCL2* rearrangement (+), no *BCL6* rearrangement |
| 089 | 60 | M | Foll NHL | MCL 5 years before | 95 | Alive | 50,XY,+X,der(1)add(1)(p36)dup(1)(q32q21),del(6)(q14-16q25-27),-10,+11,+12,t(14;18)(q32;q21),+21,+mar[cp8]/46,XY[4] | ↔ RP11-177N11 and RP11-420G9 | Telomeric 1p36 probes deleted | FISH: *IGH*-*BCL2* fusion and amplification |
| 037 | 59 | M | Foll NHL | None | 22 | Alive | 46,XY,add(1)(p36),t(6;13)(q23;q34),t(14;18)(q32;q21)[8]/46,XY[3] | ↔ RP11-177N11 and RP11-420G9 | Telomeric 1p36 probes deleted | None |
| 075 | 55 | M | Relapsed foll NHL | Dry-cleaner | 114 between initial diagnosis and relapse, 82 after relapse | Alive | 47,XY,add(1)(p36)x2,+7,add(9)(q34),t(14;18)(q32;q21),del(20)(q12)[9] | ↔ RP11-177N11 and RP11-420G9 | Telomeric 1p36 probes deleted | RT-PCR: *IGH*-*BCL2* mbr fusion (+) |
| 142 | 82 | F | Foll NHL transformed into a DLBCL | None | 14 | Alive | 46,XX,der(1)t(1;6)(p36.22;p22.2),del(12)(p13.32;p13.1),der(15)t(9;15)(q21.11;p13)[11]/46,XX[2] | ↔ RP11-177N11 and RP11-420G9 | Telomeric 1p36 probes deleted | RT-PCR: no *IGH*-*BCL2* fusion; FISH: no *BCL2* nor *BCL6* rearrangement |
| 076 | 66 | M | Foll NHL | NA | NA | NA | 47,XY,add(1)(p36),-2,add(10)(q24),-12,t(14;18)(q32;q21),+3~4mar,inc[5] | ↔ RP11-420G9 and RP11-108G8 | Telomeric 1p36 probes deleted | None |
| 115 | 53 | F | AML M1 | NA | NA | NA | 47,XX,t(1;17)(p36;q21),+4,del(9)(p21)[3]/46,idem,dic(9;18)(p13;p11),del(11)(q14q24)[5]/46,XX[5] | ↔ RP11-874A11 and RP11-56N19 | Telomeric 1p36 probes deleted | FISH: no *MLL* rearrangement |
| 110 | 65 | F | Foll NHL | None | NA | NA | 47,XX,t(1;3)(p36;p21),add(6)(q23),add(8)(p12),dup(10)(q21q23),dup(12)(q12q14),t(14;18)(q32;q21),+der(18)t(14;18)[15]/46,XX[3] | ↔ RP11-219C24 and RP11-5P18 | Telomeric 1p36 probes deleted | None |
| 080 | 52 | M | Foll NHL | NA | 30 | Alive | 47,XY,add(1)(p36),del(6)(q13q22),t(14;18)(q32;q21),+mar[6]/46,XY[1] | ↔ RP11-5P18 and RP11-91D22 | Telomeric 1p36 probes deleted | None |
| 034 | 56 | M | Relapsed AML M1 | NA | 12 between initial diagnosis and relapse, NA after relapse | NA | 46,XY,add(1)(p36.?2),i(7)(q10)[14] | ↔ RP11-79D15 and RP3-394P21 | Telomeric 1p36 probes deleted | None |
| 049 | 46 | F | Foll NHL | None | 102 | Deceased | 48-49,XX,der(1)t(1;1)(p36;q21),?der(3),+7,i(8)(q10),t(14;18)(q32;q21),+der(18)t(14;18),+mar[21]/46,XX[1] | ↔ RP3-394P21 and RP4-657E11 | Telomeric 1p36 probes deleted | FISH: *IGH*-*BCL2* (+) |
| 098 | 63 | M | CML in clonal evolution # | NA | 12 between initial diagnosis and clonal evolution, NA thereafter | NA | 46,XY,t(9;22)(q34;q11.2)[16]/46,idem,add(1)(p36)[8]/46,XY,t(9;22)(q34;q11.2),del(17)(p11)[2] | ↔ RP11-668L19 and RP11-487E1 | Telomeric 1p36 probes deleted | RT-PCR: *BCR*-*ABL1* b3a2 rearrangement (+) |
| **Telomeric rearrangements** | | | | | | | | | | |
| 030 | 29 | M | AML M2 | None | 4 | Alive | 46,XY,add(1)(p36)[7]/46,XY[4] | T1p and pan-tel retained |  | *NPM* mutation type D (+), FISH : *MLL* (-) |
| 025 | 60 | F | CEL | NA | 36 | Deceased | 46,XX,der(1)t(1;1)(p36;q12)[3]/46,XX,der(13)t(1;13)(q12;p13)[3]/46,XX,der(22)t(1;22)(q12;p13)[3]/46,XX,der(8)t(1;8)(q12;q24)[1] | T1p and pan-tel retained |  | None |
| 055 | 60 | M | AML M4 | NA | 11 | Deceased | 46,XY,der(1)t(1;1)(p36;q12)[10] | T1p and pan-tel retained |  | None |
| 090 | 48 | M | MDS | None | NA | NA | 46,XY,der(1)t(1;1)(p36;q12)[19]/46,XY[1] | T1p and pan-tel retained |  | None |
| 125 | 17 | F | FA § | FA diagnosed at age 6 | NA | NA | 46,XX,dup(1)(q11q32)[7]/46,idem,add(1)(p36)[13] | T1p and pan-tel retained |  | None |
| 131 | 13 | F | B-ALL | None | 109 | Alive | 46,XX,add(1)(p36),t(9;15)(p22;q12)[9]/46,XX[7] | T1p and pan-tel retained |  | FISH: *BCR*-*ABL1*(-), *MLL*(-), *ETV6*-*RUNX1*(-), *PAX5*(-) |
| 083 | 75 | M | MF | PV for 27 years | NA | NA | 48,XY,+8,+9[3]/48,idem,der(1)t(1;1)(p36;q12)[14]/46,XY[3] | T1p and pan-tel retained |  | None |
| 040 | 76 | F | AML | Colon cancer operated 5 years before | <1 | Deceased | 47~48,XX,add(1)(p36),-3,del(5)(q12q33),+11,add(11)(p11.2),idic(11)(p11.2),-16,der(16)?t(11;16)(q13;q24),+der(16),?r(22),+r[cp16]/46,XX[1] | T1p retained and pan-tel deleted |  | FISH: *MLL* amplification |
| 067 | 73 | M | MDS | NA | NA | NA | 42,XY,add(1)(p36),del(5)(p11p15),-14,-16,-17,-18,-20,+mar1[14]/43,idem,+mar[1]/46,XX[9] | T1p retained and pan-tel deleted |  | None |
| 099 | 77 | M | AML M2 post MDS | MDS | NA | NA | 43,XY,der(1)add(1)(p36)?del(1)(p21p31),add(4)(p16),-5,-7,add(10)(p15),add(11)(p15),-17,add(17)(q25),-18,-20,-21,+3mar[3]/40~44,XY,der(1)add(1)(p36)?del(1)(p21p31),add(3)(q26),add(4)(p16),-5,-7,add(10)(p15),add(11)(p15),-17,add(17)(q25),-18,-20,-21,+1~4mar[cp17] | T1p retained and pan-tel deleted |  | None |
| 071 | 63 | M | AML M2 post MDS | MDS | <1 | Deceased | 46-49,XY,add(1)(p36),-5,-7,add(17)(p11.?2),-18,der(20)(20pter→20q13.2 or q13.3::?::1p?12→1p36.3),+mar1,+2mar[cp16]/46,XY[2] | T1p retained and pan-tel deleted |  | RT-PCR: *TP53* exon 7 mutation |
| 122 | 76 | F | Blastoid MCL $ | CLL for 9 years | 27 | Deceased | 43,XX,add(1)(p36.3),der(3)t(3;?17)(q26;q1?2),-9,dic(12;13)(q?21;p1?),-13,-17[cp11]/46,XX[1] | T1p retained and pan-tel deleted |  | RT-PCR: *cyclin D1*(-), FISH: loss of one copy of *TP53*, no *BCL6* rearrangement |
| 123 | 62 | M | MCL $ | None | 51 | Alive | 46,XY,der(1)t(1;3)(p36;q21),t(11;14)(q13;q32)[10]/46,XY[5] | T1p retained and pan-tel deleted |  | RT-PCR: *cyclin D1*(+), FISH: *IGH*-*CCND1*(+), *BCL6* 3 copies |
| 065 | 55 | F | TCL * | NA | NA | NA | 46,X,der(X)t(X;1)(q28;q21),add(4)(q21),del(6)(q12q22),+7,dic(7;20)(p21;q13.3),add(16)(q22),+mar[4]/48~52,idem,+X,add(1)(p36),+5,+dic(7;20)(p21;q13.3),+0~2mar[cp4]/46,XX[7] | T1p retained and pan-tel uninterpretable |  | FISH: *IGH*-*CCND1*(-), *IGH*-*BCL2*(-) |
| 031 | 66 | M | PV | None | 69 | Alive | 46,XY,add(1)(p36)[2]/46,XY[8] | T1p retained and pan-tel uninterpretable |  | None |

Abbreviations : Pt = patient, yrs = years, CLL = chronic lymphocytic leukemia, AML = acute myeloid leukemia, AML M0 = minimally differentiated acute myeloblastic leukemia, AML M1 = acute myeloblastic leukemia, without maturation, AML M2 = acute myeloblastic leukemia, with granulocytic maturation, AML M3 = acute promyelocytic leukemia, AML M4 = acute myelomonocytic leukemia, AML M5 = acute monoblastic leukemia (M5a) or acute monocytic leukemia (M5b), AML M6 = acute erythroid leukemia, ALL = acute lymphoblastic leukemia, MDS = myelodysplastic syndrome, RAEB = refractory anemia with excess blasts, RARS = refractory anemia with ring sideroblasts, RTC = refractory thrombocytopenia, CML = chronic myelogenous leukemia, PV = polycythemia vera, M+ = metastatic, CEL = chronic eosinophilic leukemia, FA = Fanconi's anemia, MF = myelofibrosis, MCL = mantle cell lymphoma, TCL = T-cell lymphoma, PTCL = peripheral T-cell lymphoma, NHL = non-Hodgkin lymphoma, Foll = follicular, DLBCL = diffuse large B-cell lymphoma, AUL = acute undifferentiated leukemia, LPS = B-cell lymphoproliferative syndrome, CTH = chemotherapy, RTH = radiotherapy, +21 = trisomy 21, BC = breast cancer, NA = not available, F = female, M = male, ↓ = within, ↔ = between, T1p = subtelomeric Vysis TelVysion 1p probe, pan-tel = pan-telomeric probe Star*FISH©

* There were one case of T-cell lymphoma (case 121) with a balanced translocation involving 1p36.22, and 3 cases of T-cell malignancies with add(1)(p36) at a very distal level, ranging from 1p36.33 to 1p36.31 (cases 065, 136 and 153). 1p36 alterations in lymphomas are not restricted to B-cell lineage. Large cell transformation in mycosis fungoides is known to be associated with 1p36 gains {Prochazkova, 2007 #25}. Array-CGH analysis previously showed losses on 1p in 38% of a series of 34 primary cutaneous TCL, with a wide minimal region of deletion at 1p31p36 {Mao, 2002 #35}.

# There was one case of CML in clonal evolution with a deletion spanning the distal 21 Mb of 1p36. Disease progression in CML is known to be accompanied by 1p36 deletions {Brazma, 2007 #24} {Mori, 1998 #37}, in addition to cryptic deletions of *PRDM16* {Deluche, 2008 #59}.

§ Case 125 in the group of telomeric rearrangements was a Fanconi anemia (FA). The gain of the long arm of chromosome 1 is found in 37.5% of FA patients with normal bone marrow {Quentin, 2011 #56}. FA is a recessive genetic disease characterized by increased spontaneous and induced chromosome instability. Accelerated telomere shortening and increased chromosome end fusions have been observed in FA {Callen, 2002 #51}.

$ The only 2 MCL in our study (cases 122 and 123) had a very telomeric 1p36 deletion (the sub-telomeric probe was retained but not the pan-telomeric one). In one case, we observed a telomeric deletion limited to 1p. In the other case, the pantelomeric signals were undetectable on all chromosomes in the abnormal metaphases at our resolution limit of 3.5 kb, while the signals remained present in the normal metaphases, reflecting the genetic instability of the malignant clone. Genomic instability can be acquired. It is one of the common features of MCL {Perez-Galan, 2011 #47}, with telomere length shorter in patients with MCL than in controls {Cottliar, 2009 #50}.
